# Intestinal helminthiasis survey with emphasis on schistosomiasis in Koga irrigation scheme environs, northwest Ethiopia

**DOI:** 10.1371/journal.pone.0272560

**Published:** 2022-08-08

**Authors:** Zemenu Tamir, Abebe Animut, Sisay Dugassa, Araya Gebreselassie, Aster Tsegaye, Tesfu Kassa, Tadesse Eguale, Tadesse Kebede, Yohannes Negash, Zeleke Mekonnen, Berhanu Erko

**Affiliations:** 1 Department of Medical Laboratory Sciences, College of Health Sciences, Addis Ababa University, Addis Ababa, Ethiopia; 2 Aklilu Lemma Institute of Pathobiology, Addis Ababa University, Addis Ababa, Ethiopia; 3 Department of Zoological Sciences, College of Natural and Computational Sciences, Addis Ababa University, Addis Ababa, Ethiopia; 4 Department of Microbiology Immunology and Parasitology, School of Medicine, Addis Ababa University, Addis Ababa, Ethiopia; 5 School of Medical Laboratory Sciences, Institute of Health, Jimma University, Jimma, Ethiopia; Institute of Cytology and Genetics, RUSSIAN FEDERATION

## Abstract

**Background:**

Distribution of schistosomiasis is more focal due to spatial heterogeneities in intermediate host snail dynamics and water contact behavior of humans. This makes the search for new transmission foci of schistosomiasis and its connection with malacologically receptive water bodies essential for effective control of its transmission. This study was intended to assess the prevalence of intestinal helminth infections among schoolchildren and *Schistosoma mansoni* transmission in Koga irrigation scheme surroundings, northwest Ethiopia.

**Materials and methods:**

Cross-sectional parasitological and malacological surveys were conducted in three schools and nearby water bodies, respectively around Koga irrigation scheme. Stool specimens were collected from 421 randomly selected schoolchildren and microscopically examined using Kato-Katz and formol-ether concentration methods. Malacological surveys were carried out and the identified *Biomphalaria pfeifferi* snails were screened for schistosome infection. Swiss albino mice were exposed to schistosome cercariae shed by *Biomphalaria pfeifferi* for definite identification of *Schistosoma* species.

**Results:**

Among the examined schoolchildren, 22.6% (95% CI: 18.7%-26.9%) were positive for at least one intestinal helminths species. *Ascaris lumbricoides* was the most frequent intestinal helminth detected among forty (9.5%) children. *Schistosoma mansoni* was detected among 4.8% (95% CI: 2.9%-7.2%) of children and its prevalence was significantly higher among male children (p = 0.038) and those attending in Mengesha Jemberie Primary School (p = 0.044). *Biomphalaria pfeifferi* snails were identified in water bodies in close proximity to Mengesha Jemberie and Wotete Abay Primay schools. *Schistosoma mansoni* adult worms were harvested after exposure of mice to cercariae shed from *Biomphalaria pfeifferi* snails collected from water bodies nearby Mengesha Jemberie Primary School.

**Conclusions:**

*Schistosoma mansoni* infection of schoolchildren, findings of schistosome infected snails and establishment of mice infection confirm that transmission is taking place in the study areas. Hence, snail control and other measures such as provision of sanitary facilities and health education are recommended.

## Introduction

Schistosomiasisis is a chronic debilitating parasitic disease caused by blood flukes of the genus *Schistosoma* [1]. *Schistosoma haematobium*, *S*.*mansoni*, *S*. *japonicum*, *S*.*guineensis*, *S*.*intercalatum* and *S*.*mekongi* are the etiologic agents of schistosomiasis. Among them, the former two are the most important human infections in sub-Saharan Africa causing urogenital and intestinal schistosomiasis, respectively [2].

Schistosomiasis ranks second to malaria among parasitic diseases in terms of socio-economic and public health importance in tropical countries [3, 4]. The disease is endemic in 78 resource constrained countries [5], and affects close to 240 million people worldwide [6]. Sub-Saharan Africa contributes 93% of the global cases of schistosomiasis and is used as one of the indicators of poverty [7, 8]. Similarly, soil-transmitted helminth(STHs) infections including *Ascaris lumbricoides*, hookworms (*Ancylostoma duodenale and Necator americanus*) and *Trichuris trichiura* are among the prevailing and major public health problems in sub–Saharan Africa; where 80% of the 1.5 billion globally infected people reside [9, 10].

Schistosomiasis and soil-transmitted helminthiasis remain significant public health problems in Ethiopia [11]. Ethiopia ranked 13^th^ highest burdened country for both diseases among 40 African countries [12] with an estimated thirty-six and five million infected individuals, respectively, often in impoverished and remote areas [13]. National mapping surveys conducted in Ethiopia indicated widespread distribution of STHs and distinct regional distribution of *S*.*mansoni* whereas a more restricted distribution of *S*. *haematobium*, primarily to low land areas of the country [14].

School-age children (SAC), pregnant women and individuals who frequently contact fresh water bodies are most vulnerable to schistosome infections [15]. Schistosomiasis in developing countries is more abundant among SAC due to their frequent water contact behavior and immature acquired immunity [11, 16, 17]. Infected children faced stunted growth, anemia and poor cognitive performance [18]. Hence, the control programs of STHs and schistosomiasis are mostly targeted at SAC.

Open field defecation practices, presence of host snails and free swimming cercaria infested water bodies expose people to schistosomiasis [1, 15]. As a result of variations in the host snail dynamics and human water contact behaviors, prevalence and intensity of schistosome infections varies even within a small area, from one village to another [1, 19]. Despite substantial successes in minimizing the disease burden and egg intensity among treated SAC [20], the schistosomiasis control program in Ethiopia has several challenges. This could partly result from incomplete mapping, re-infection, program reliance on mass drug administration, and classification of *S*. *mansoni* transmission areas at district level while the transmission is highly focal [15, 20, 21]. Hence, the search for new transmission foci of schistosomiasis and its connection with water bodies is essential for effective control of schistosomiasis transmission.

Reports from health facilities in Dangla and Merawi towns indicated occasional detection of *S*. *mansoni* among patients visiting the local health facilities and majority of them were from villages where small rivers are found. In addition, Koga irrigation scheme which could be potential receptive site for *Schistosome* snails is found around Merawi and Wotet Abay areas, North Mecha district. Hence, this study was conducted to determine the prevalence of intestinal helminth infections among schoolchildren attending schools in the area and *S*. *mansoni* transmission in nearby school water bodies.

## Method and materials

### Study design and area

**Fig 1:**
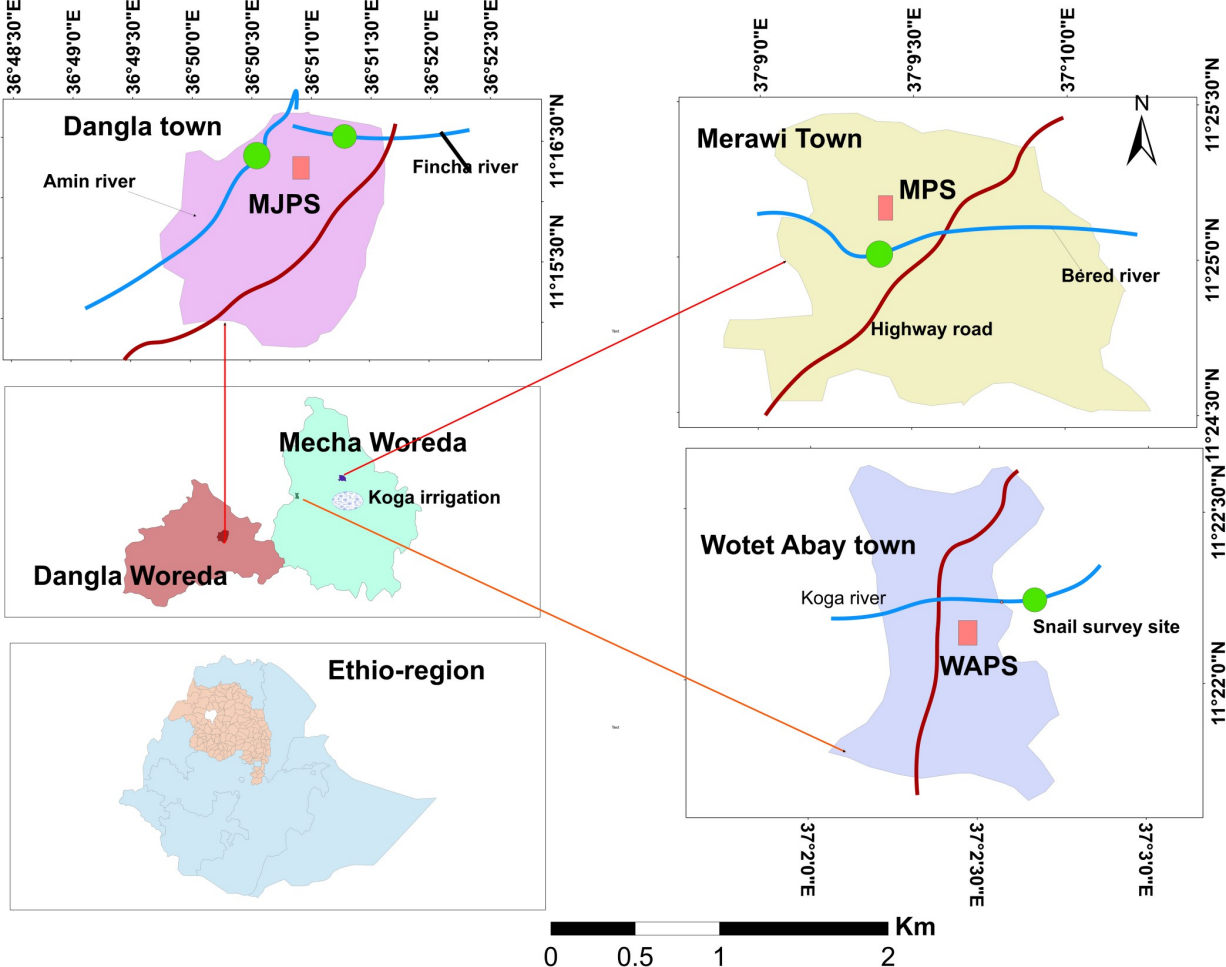
Map of the study area and data collection sites.

A cross-sectional parasitological survey was conducted among schoolchildren attending Mengesha Jemberie, Merawi and Wotet Abay Primary Schools, northwestern Ethiopia from May-June, 2021. Malacological surveys were also carried out along water bodies found in close proximity to the respective schools (Fig 1). These schools were purposively selected because of the presence of rivers and perennial streams in the area which would create suitable habitat for snail intermediate hosts.

Mengesha Jemberie Primary School (MJPS) is found in Dangla town, Amhara Regional State, northwest Ethiopia; about 480 Kilometers away from Addis Ababa. The town is located at 11°16′N 36°50′E with an elevation of 2137 meters above sea level. Amin River and Finch Stream are found in close proximity to the school to which schoolchildren have had frequent contacts.

Merawi Primary School (MPS) is found in Merawi town and Wotet Abay Primary School (WAPS) in Wotet Abay town. Merawi and Wotet Abay towns are found in Semien Mecha District, West Gojjam Zone, Amhara Regional State, northwest Ethiopia. Merawi town is located about 30 kilometers south of Bahir Dar and 525 kilometers away from Addis Ababa. Specifically, the town is located lying at the 11°24′31″N 37°9′39″E with an elevation of 1901 meters above sea level. Wotet Abay town is located about 12 kilometers to the south of Merawi town at the coordinates of 11°22’11"N 37°2’30"E with an elevation of 1905m above sea level east to the Gilgel Abay River bank. Bered and Koga rivers are found in close proximity to Merawi and Wotet Abay Primary schools, respectively, where schoolchildren have had frequent contacts. In addition, Koga irrigation scheme is found in Merawi and Wotet Abay area in Semien Mecha district laying between 11°20ʹ to 11°32ʹ North and 37°02ʹ to 37°11ʹ East (Fig 1).

### Sample size and sampling technique

Using single population proportion sample size determination formula $n=\frac{{{(Z}_{\frac{\alpha }{2}})}^{2}(P(1‐P)}{d^{2}}$, assuming an estimated proportion P = 50% of *S*.*mansoni* infection among schoolchildren since there was no similar study in the study area, $Z_{\frac{\alpha }{2}}$ = 1.96 at 95% confidence level, margin of error (d) of 5% and non- response rate of 10%, the sample size was determined to be 422. The number of children enrolled in the study from each school were determined in proportion to size based on the number of SAC attending in each school. Accordingly, 126, 131, and 164 schoolchildren were enrolled from Mengesha Jemberie, Merawi and Wotet Abay Primary Schools, respectively. In each school, the total required sample was distributed to each class (n_c_) and the students were selected using systematic random sampling technique taking the class register as a sampling frame, listing the students as: 1,2,3,…..N, and interval(k). The interval k was determined by using the formula, $k=\frac{N}{n_{c}}$; and the first participant was selected by lottery method from the list 1 to k.

### Data collection procedure and laboratory analysis

Structured pretested questionnaire S1 File was used to collect data on socio-demographic and associated risk factors of schistosomiasis among children. The collected data includes children profile such as age, sex, grade level, residence, latrine availability in schools and practice of use of children, shoes wearing habit and type of shoes, de-worming status and water contact behaviors.

For parasitological surveys, selected children were given a clean plastic stool cup with wooden applicator stick and instructed to bring about 2 grams of their own stool. Two Kato-Katz (KK) slides (using 41.7 mg template) were prepared from single stool specimen of each child and the remaining portion of the stool specimen was preserved in 10% formalin solution for examination by formol-ether concentration technique (FECT). The prepared slides and preserved specimens were transported to the Medical Parasitology Laboratory of Aklilu Lemma Institute of Pathobiology, Addis Ababa University, within five days of specimen collection. The KK slides were stored in refrigerators until examination. The KK thick smear technique was used for detection and quantification of *S*. *mansoni* eggs and for detection of other intestinal helminth species [22]. The FECT was used for detection of *S*. *mansoni* eggs and other intestinal helminth species. However, examination for hookworm parasite was made from the 10% formalin preserved stool specimen only. Collection of stool specimen, transportation, processing, storage, examinations and all other laboratory procedures were conducted following the recommended standard operational procedures to ensure quality. Stool specimen was collected, processed and examined by two trained medical laboratory professionals cross-checked by a third more experienced professional.

A stool specimen from each child was considered positive for *S*. *mansoni* or other intestinal helminth species if eggs were detected by either of the two or both methods. A specimen was considered negative for *S*. *mansoni* or other intestinal helminths species if no egg of these parasites were detected from two KK thick smear slides and FECT slide, except specimens collected from Mengesha Jemberie Primary School which were processed using KK methods only. The intensity of *S*. *mansoni* infection(expressed in eggs per gram of stool, EPG) was determined by multiplying the mean of eggs counted from both 41.7 mg template KK slides by a factor of 24 [23].The infection intensity of *S*.*mansoni* was classified as light (1–99 EPG), moderate (100–399 EPG) and heavy (> 400 EPG) infection [24]. The community risk of schistosomiasis transmission was leveled based on the prevalence of schistosomiasis among schoolchildren as high risk (> 50% prevalence), moderate risk (20–50% prevalence) and low risk community (< 20% prevalence) [15, 24].

### Malacological survey and mice infection

Surveys for intermediate host snails were conducted at water contact sites in the small rivers close to the schools to determine presence of snails and schistosome infection in the snails. The snails were collected from May to June, 2021 from the water bodies of Amin River and Fincha Stream which are found in close proximity to Mengesha Jemberie Primary School; Bered and Koga Rivers which are at close proximity to Merawi and Wotet Abay Primary Schools, respectively. Schoolchildren attending the respective schools had frequent contacts with these water bodies. Koga irrigation dam is constructed on the upper bank of Koga River. The snails were collected from the human water contact sites by handpicking using forceps and gloves. The collected snails were then placed in plastic buckets containing water and aquatic weeds, and transported to the Medical Parasitology Laboratory of Aklilu Lemma Institute of Pathobiology in Addis Ababa.

The collected snails were identified to species level based on shell morphology [25]. The identified *B*. *pfeifferi* were examined for natural trematode infections by shedding method. Briefly, the snails were placed individually in the shedding vials containing aged water and then exposed to electric light for about one hour to induce cercariae shedding. The shed cercariae were identified to the *Schistosoma* genus level using tail morphology and swimming behavior[25].

Laboratory bred Swiss albino mice were exposed to the cercariae shed from the snails for definite identification of schistosome species based on egg or adult worm morphology. This was done by immersing the tail and legs of the mouse in a beaker containing cercariae and aged water for 30 minutes to establish infection. The mice were sacrificed after 6 weeks of exposure and adult *Schistosoma* worms were harvested and identified from the mesenteric veins [26]. Definite identification of the schistosome parasites were made based on eggs and the adult worm morphology [27]. Survey for intermediate host snails, identification of snail species and Schistosme genus cercaria, mice infection, sacrificing, adult worm harvesting and identification were made by experienced professionals and a senior researcher.

### Statistical analysis

Data were cleaned and double entered into Epi Info version 3.4.3 software to reduce clerical errors and inconsistencies. The entered data was exported to Statistical Package for Social Sciences (SPSS) version 25 statistical software (SPSS INC, Chicago, IL, USA) and analyzed. Prevalence of *S*. *mansoni* and other intestinal helminths infection were estimated by dividing the number of schoolchildren diagnosed positive by the total number of schoolchildren examined. Overall mean of *S*. *mansoni* EPG of stool specimens were determined by dividing the sum of all the means of EPG of the two KK thick smears of each child by the number of positively diagnosed schoolchildren to estimate the intensity of *S*. *mansoni* infection.

Chi-square and Fisher’s exact test were used to assess the relationship between prevalence of *S*. *mansoni* and other helminths infections with independent variables such as: sex, age, attending school, residence and grade level. Bivariable and multivariable logistic regression models were used to identify factors that contribute to intestinal schistosomiasis. Odds ratio (OR) with 95% confidence interval (CI) was used to measure the strength of statistical association. P value *<*0.05 was used to indicate statistical significance.

### Ethical consideration

This study was conducted after the protocol was reviewed and approved by the Institutional Review Board (IRB) of Aklilu Lemma Institute of Pathobiology, Addis Ababa University. Permission to conduct the study was also obtained from the health offices and educational offices of Dangla district and Semien Mecha district as well as principals of each school after having thorough discussion on the procedures and purpose of the study. Before commencement of data collection, written consent was obtained from parents/guardians of children and the participating children also gave their assent for participation.

## Results

A total of 421 SAC who had provided sufficient stool specimen for diagnosis of intestinal helminth infections were involved. The mean ± standard deviation (Sd) age of children was 10.9±2.16 years (range: 6–15 years) and 55.8% of them were males. The distribution of the included children was 29.9%, 31.1% and 39% from Mengesha Jemberie, Merawi and Wotet Abay Primary Schools, respectively. About 94.3% of children reported that latrine was available to the family and that was used consistently, Table 1.

**Table 1:** Socio-demographic characteristics of the study participant schoolchildren in northwest Ethiopia, n = 421.

Characteristics	Category	Number(N)	Percentage (%)
**Sex**	Female	186	44.2
	Male	235	55.8
**Age group (Year)**	6–10	199	47.3
	11–15	222	52.7
**Residence**	Urban	348	82.7
	Rural	73	17.3
**Schools**	Mengesha Jemberie Primary School	126	29.9
	Merawi Primary School	131	31.1
	Wotet Abay Primary School	164	39.0
**Grade level**	1–4	277	65.8
	5–8	144	34.2
**Latrine Availability**	No	24	5.7
	Yes	397	94.3

### Prevalence of *Schistosoma mansoni* and intestinal helminths infections

In this study, among 421 SAC who delivered sufficient stool specimens, 95 children (22.6%, 95% CI: 18.7%-26.9%) were infected with one or more of intestinal helminths parasites. *Schistosoma mansoni* infection was detected among 20 children (4.8%, 95% CI: 2.9%-7.2%): as single infection among 13 children and co-infection or triple infection with other intestinal helminths among 7 children. Other intestinal helminth infections were detected in 82 (19.5%, 95% CI: 15.8%-23.6%) schoolchildren. *Ascaris lumbricoides* was the most frequent soil transmitted helminth found among 40 schoolchildren (9.5%) followed by hookworm (5.4%), which was identified from FETC preparations among Merawi and Wotet Abay Primary School students only; and *T*. *trichiura* (2.9%). Eleven schoolchildren were co-infected with intestinal helminths whereas one student harbored triple infection as detailed in Table 2.

**Table 2:** Distribution of intestinal helminth infections among the study participant schoolchildren in northwest Ethiopia, N = 421.

Parasite Species	Infected children, n*(%)
***S*. *mansoni***	13(3.1)
***A*. *lumbricoides***	30(7.1)
**Hookworm** **	14(4.7)
***T*.*trichiura***	10(2.4)
***H*.*nana***	13(3.1)
***E*.*vermicularis***	3(0.7)
***A*.*lumbricoides* and *S*.*mansoni***	4(1)
***Hookworm and S*.*mansoni***	2(0.7)
***A*.*lumricoide* and *H*.*nana***	3(0.7)
***A*.*Lumricoide* and *T*.*trichiura***	2((0.5)
***A*.*lumbricoides* and *H*.*nana* and *S*.*mansoni***	1(0.2)

*n = frequency

**hookworm examination was made from FETC preparations (Merawi and Wotet Abay Primary school students), n = 295

Prevalence of *S*. *mansoni* infection showed variability among schools ranging from 8.7% in Mengesha Jemeberie Primary School to 2.3% in Wotet Abay Primary School *(p = 0*.*037)*. *Schistosom mansoni* infection was relatively higher among male children than females (6.8% vs 2.2%), p = 0.045; urban children than rural children (5.2% vs 2.7%), p = 0.558; and among relatively matured children than the youngers (5.4% vs 4%), P = 0.066. Although the differences were not significant, prevalence of other helminths also showed variability among school types, residence, gender, age group and grade level of schoolchildren following the same trend with schistosomiasis, except that the schoolchildren from rural villages had higher prevalence of intestinal helminths infections than the urban ones (Table 3).

**Table 3:** Distribution of *S*. *mansoni* and other intestinal helminths among study participants with respect to socio-demographic variables, northwest Ethiopia, N = 421.

Variable		*S*. *mansoni* infection		Other intestinal helminths infection	
		No, n(%)	Yes, n(%)	No, n(%)	Yes, n(%)
**School**	Mengesha Jemberie Primary School	115(91.3)	11(8.7)	100(79.4)	26(20.6)
	Merawi Primary School	128(97.7)	3(2.3)	105(80.2)	26(19.8)
	Wotet Abay Primary School	158(96.3)	6(3.7)	134(81.7)	30(18.3)
	P value	0.037		0.762	
**Sex**	Male	219(93.2)	16(6.8)	187(79.6)	48(20.4)
	Female	182(97.8)	4(2.2)	152(81.7)	34(18.3)
	P-value	0.045		0.668	
**Age in years**	6–10	191(96)	8(4)	164(82.4)	35(17.6)
	11–15	210(94.6)	12(5.4)	175(78.8)	47(21.2)
	P-value	0.066		0.442	
**Grade level**	1-4^th^	268(96.8)	9(3.2)	228(82.3)	49(17.7)
	5-8^th^	133(92.4)	11(7.6)	111(77.1)	339(22.9)
	P-value	0.077		0.248	
**Residence**	Rural	71(97.3)	2(2.7)	55(75.3	18(24.7)
	Urban	330(94.8)	18(5.2)	284(77.6)	64(18.4)
	P-value	0.549^a^		0.286	

^a^P-value was calculated using Fisher’s exact test

In this study, the mean intensity of *S*. *mansoni* infection among infected SAC was 121.2 EPG of stool specimen examined. Among the infected children, 55% and 45% had light and moderate intensity of *S*. *mansoni* infection, respectively, but none had heavy infection.

### Factors associated with *Schistosoma mansoni* infection

For assessment of associated independent risk factors of *S*. *mansoni* infection, bivariable and multivariable logistic regression models were computed. Accordingly, sex of schoolchildren was identified as determinant factor for intestinal schistosomiasis. Male schoolchidren were 3.36 times more likely to be infected with intestinal schistosomiasis than their female counterparts (AOR = 3.36, 95% CI [1.07, 10.6], P = 0.038). The likelihood of schoolchildren to acquire *S*. *mansoni* infection was also variable among the different schools. Children attending school at Mengesha Jemberie Primary School were 4.42 times more likely to get *S*. *mansoni* infection than children attending school at Merawi Primary School (AOR = 4.42, 95% CI [1.04,8.7], p = 0.044). Similarly, the study also revealed that those children who use to bath, swim or having frequent contact with the nearby school river/stream water have had 4.52 times increased risk of intestinal schistosomiasis than those who did not(COR = 4.52, 95% CI[1.03,19.75], p = 0.045).

There was increased likelihood of *S*. *mansoni* infection among schoolchildren within 11–15 years of age, at advanced grade level, those wearing open shoes or do not wear shoes and living in the urban areas. These observations indicated the likelihood that students acquired the infection from the nearby school rivers or streams. The study also showed that there was no difference of schistosomiasis infection among schoolchildren who took school-based deworming before 1 year and those who did not take (p = 0.972) (Table 4).

**Table 4:** Factors associated with occurrence of intestinal schistosomiasis among schoolchildren in northwest Ethiopia (n = 421).

Characteristics		Schistosomiasis		COR[95% CI]	P-value	AOR[95%CI]	P-value
		No, n(%)	Yes, n(%)				
**Sex**	Female	182(97.8)	4(2.2)	1	0.034	1	0.038
	Male	219(93.2)	16(6.8)	3.32[1.09–10.12]		3.36[1.07,10.6]	
**Age group**	6–10	191(96)	8(4)	1	0.56	1	0.325
	11–15	210(94.6)	12(5.4)	1.36[0.55,3.41]		2.94[0.34,8.3]	
**Residence**	Rural	71(97.3)	2(2.7)	1	0.383	1	0.558
	Urban	330(94.8)	18(5.2)	1.94[0.44,8.53]		3.77[0.65,13.08]	
**School^@^**	MJPS	115(91.3)	11(8.7)	4.08[1.11,14.99]	0.037	4.42[1.04,8.7]	0.044
	MPS	128(97.7)	3(2.3)	1	-	1	-
	WAPS	158(96.3)	6(3.7)	2.52[0.91,7.01]	0.077	5.48[0.65,14.5]	0.117
**Grade level**	1–4	268(96.8)	9(3.2)	1	0.051	1	0.226
	5–8	133(92.4)	11(7.6)	2.46[0.97,6.1]		4.1[0.42,12]	
**Type of shoes**	Closed	87(97.8)	2(2.2)	1	0.234	1	0.133
	Open	313(94.6)	18(5.4)	2.46[0.56,10.8]		3.28[0.69,5.5]	
**Deworming before 1 yr**	Yes	239(95.2)	12(4.8)	1	0.97		
	No	162(95.3)	8(4.7)	1.02[0.41,2.54]			
**Water contact**	No	134(98.5)	2(1.5)	1	0.045	1	0.148
	Yes	267(93.7)	18(6.3)	4.52[1.03,19.75]		3.14[0.66,10.8]	
**Latrine availability**	yes	379(95.5)	18(4.5)	1	0.403	1	0.35
	No	22(91.7)	2(8.3)	1.91[0.42,8.78]		2.4[0.38,15.2]	

^@^MJPS = Menegesha Jemberie Primary School, MPS = Merawi Primary School, WAPS = Wotet Abay Primary School

^#^Crude odds ratio

^$^ adjusted odds ratio

### Malacological surveys and mice infection

During the malacological surveys from May to June 2021, *Biomphalaria pfeifferi* snails were collected from Amin River and Fincha Stream located in close proximity to Mengesha Jemberie Primary School and from the Koga River which is located in close proximity to Wotet Abay Primary School to which students have frequent contacts. A total of 2, 33, and 3 *B*.*pfeifferi* snails were collected from Fincha Stream, Amin and Koga Rivers respectively. Other snail species such as *Bulinus forskali* and *Lymnaea natalensis* were also collected form Fincha Stream, Koga, and Bered Rivers.

Twenty-seven *B*. *pfeifferi* snails collected from Amin River were examined for natural trematode infections by the shedding method whereas snails collected from Koga River and Fincha Stream died before examination. Among the 27 *B*. *pfeifferi* snails, two shed cercariae. Ten laboratory bred mice were exposed to the cercariae shed from the snails. Among the exposed mice, three mice died within 6 weeks of maintenance and the remaining were sacrificed after 6 weeks of exposure and the number of adult schistosome worms were counted. A total of 30 adult *S*.*mansoni* worms from two mice (4 copula and 6 males from mouse 1 and 16 males from mouse 2) were harvested.

During the snail survey, physical characteristics of the water bodies of the rivers and streams of the survey sites were assessed. Accordingly, Amin and Bered Rivers were turbid, muddy, medium in size, and slow flowing; they were covered with weeds, algae, garbage plastics, clothes and fallen leaves. Koga River was large, fast flowing, clear and have limited vegetation, weeds and algae in the lower bank whereas in the upper bank relatively wide spread, slow flowing, and have abundant algae and sea weeds. Fincha Stream, on the other hand, was clear, slow flowing, very small and largely covered by sea weeds, algae, other garbage such as plastics, clothes and other wastes.

## Discussion

This study aimed to determine prevalence of intestinal helminths infections among schoolchildren and transmission of intestinal schistosomiasis in water bodies in close proximity around the schools in Koga irrigation scheme area, northwest Ethiopia. Overall, 4.8% and 19.5% of schoolchildren who participated in this study were infected with *S*. *mansoni* and other intestinal helminths, respectively. Different literatures indicated that helminths infections in general and schistosomiasis in particular are associated with chronic under nutrition and anemia leading to stunted growth and impaired cognitive development in children resulting impaired learning abilities [28–30]. The prevalence of intestinal helminths and *S*. *mansoni* in this study were lower than the prevalence of STHs and *S*. *mansoni* among schoolchildren in Amhara region reported by Nute et al which was 36.4% and 6.4%, respectively [12]. The lower result in the current study might be due to the control practice through the nationwide mass drug administration program among schoolchildren since 2015. Similarly, the finding of this study is lower than studies in northeastern Ethiopia [31], southwestern Ethiopia [32] and south-central Ethiopia [15]. However, our finding is higher than the report of Leta et al on the status of STHs and schistosomiasis among school aged children in Amhara region in their national mapping survey [14].

Generally, the transmission of schistosomiasis is highly focal and directly related to the geographical range of their intermediate snail hosts which could be influenced by climate, altitude, rainfall, water chemistry and aquatic vegetation [19, 33]. Presence of *B*. *pfeifferi* snails and the water contact behavior of individuals such as bathing, swimming and fishing activities determine the transmission of *S*.*mansoni* [34]. In this study, the prevalence of *S*. *mansoni* showed significant difference among the assessed schools: highest in Mengesha Jemberie Primary School and lowest in Merawi Primary School. This finding revealed that schoolchildren attending at Mengesha Jemberie Primary School were 4.42 times more likely to get infected by *S*. *mansoni* than those attending in Merawi Primary School. The increased prevalence of schistosomiasis in this school could be attributed with the presence of the intermediate host *B*. *pfeifferi* snails in the water bodies of Amin River and Fincha Stream as demonstrated during our snail survey which are in close proximity to the school and students have frequent contact. Moreover, the collected *B*. *pfeifferi* snails from the Amin River were infested with *S*. *mansoni* and its infectiousness were demonstrated in the laboratory bred mice.

Collecting *B*. *pfeifferi* snails in Koga River and finding of *S*. *mansoni* among schoolchildren of Merawi and Wotet Abay Primary Schools in this study have implications for risk of introducing schistosomes into Koga irrigation scheme which could contribute for transmission of schistosomiasis in the community. The current study had also showed that those schoolchildren who had reported having frequent contacts to the nearby school water bodies had more than four times increased risk of intestinal schistosomiasis than those who did not have. In line with the current study, a study conducted in Wondo district, West Arsi Zone, Oromia Regional State, Ethiopia reported that children who had swimming habit in rivers were almost 10 times more likely to be infected with *S*. *mansoni* than those who did not have [35]. Similarly, Alebie et al from Sanja, northwest Ethiopia showed that schoolchildren who had swimming habit, wash cloths and bathe in the rivers had more than three times higher risk of *S*. *mansoni* infection than those who did not [36].

Studies have revealed that gender has been shown to impact likelihood of infection and reinfection by schistosomiasis [37]. The current study has revealed that intestinal schistosomiasis infection is significantly higher in male schoolchildren than females, demonstrating males were more than 3 times more likely to be infected by *S*. *mansoni* than their female counterparts. This was in agreement with previous studies in Ethiopia [21, 38–40]. The higher infection status of male schoolchildren in this study might be attributed to the increased water contact habit of males in nearby school water bodies (73.6%) than females (60.2%).

Previous studies have also demonstrated that the prevalence of schistosomiasis among children tended to rise with age [31]. Although the difference was not statistically significant, this study showed that children within 11–15 years of age were at higher risk of schistosoma infection than 6–10 years (5.4% Vs 4%). This was in agreement with reports by Bekana et al from southwest Ethiopia [32] and Tiruneh et al from south-central Ethiopia [15] revealing higher infection status relatively among elder schoolchildren but not with Wubet et al and Worku *et al* from northwest Ethiopia [38, 41] which reported relatively higher prevalence of intestinal schistosomiasis among children who were younger and of low grades. This could be attributed by a relatively increased water contact behavior of elder children than their younger counterparts, 75.2% and 59.3%, respectively.

Studies from Ethiopia have shown that schistosome and STHs infections remain significant health problems among SAC despite repeated yearly MDA campaigns [42, 43]. A study from northwest Ethiopia reported that *S*.*mansoni* infection prevalence among SAC has decreased from 20.3% before the MDA initiation to 8.8% after MDA initiation, despite the prevalence remaining constant during the MDA program [44]. Similarly, a study conducted in Sanja town, northwest Ethiopia reported a higher rate of *S*.*mansoni* re-infection among schoolchildren after six months of effective treatment [21]. On the other hand, a study conducted in Northern Ethiopia revealed a significant and consistent decrease in the prevalence of schistosomiasis during the campaign years of a comprehensive program combining intensive health education, water sanitation and preventive deworming (44.7% before initiation of program to 4.8% at the end of the program) [17]. The present study showed that there was insignificant difference of intestinal schistosomiasis infection among schoolchildren who took school-based deworming before a year (4.8%) and those who did not (4.7%), (P = 0.972). This may indicate an ongoing re-infection among the study participants which could be attributed by the presence of intermediate host snail infested water bodies in close proximity to schools to which schoolchildren have frequent contact. Hence, this would suggest for integrated control of schistosomiasis in addition to the deworming programs.

The findings of this study should be considered alongside its limitations. First, the parasitological examination of Kato-katz smears were not conducted within 24 hours of collection which could lead to underestimation of the reports. Secondly, the malacological surveys were conduct only in one round hence unable to completely rule out possible transmission of schistosomiasis in the areas where *B*. *pfeifferi* snails were not found in the surveyed rivers during the survey period. On top of these, the study was conducted in a relatively small sample size, 421 schoolchildren, which was also coupled with lower positivity rate of schistosomiasis among the study participants, 4.8%. When these fewer cases distributed to the different categories of independent variables, it might not able to predict the outcome variable precisely hence a wide confidence interval was observed around the odds ratios.

## Conclusions

This study showed lower prevalence of *S*. *mansoni* infection among schoolchildren in the study area, hence a lower transmission risk in the community. However, *Schistosoma mansoni* infection of schoolchildren, finding of schistosome infected snails in nearby schools water bodies, establishment of mice infection and harvesting adult worms confirm that transmission is taking place in the study areas. Moreover, collecting *B*.*pfeifferi* snails in Koga River and finding infected children in Merawi and Wotete Abay Primary Schools posed risk of introduction of schistosomes into Koga irrigation scheme, which could implicate transmission of schistosomiasis in the community. Hence, snail control, preventive chemotherapy and other measures such as provision of sanitary facilities and health education are recommended.

## Supporting information

S1 File Questionnaire for a study on intestinal helminthiasis survey with emphasis on schistosomiasis in Koga irrigation scheme environs, northwest Ethiopia. (PDF)

S2 File Amharic version of questionnaire. (PDF)

S3 File Ethical clearance letter. (PDF)

S4 File SPSS data file for a study on intestinal helminthiasis survey with emphasis on schistosomiasis in Koga irrigation scheme environs, northwest Ethiopia. (SAV)

S1 Table Association of sex and age with water contact characteristics of study participant schoolchildren in northwest Ethiopia, N = 421. (PDF)

S1 Fig Picture of snail collection site water bodies for a study on intestinal helminthiasis survey with emphasis on schistosomiasis in Koga irrigation scheme environs, northwest Ethiopia. (TIFF)

S2 Fig Picture of snail collection site water bodies for a study on intestinal helminthiasis survey with emphasis on schistosomiasis in Koga irrigation scheme environs, northwest Ethiopia. (TIFF)

S3 Fig Picture of snail collection site water bodies for a study on intestinal helminthiasis survey with emphasis on schistosomiasis in Koga irrigation scheme environs, northwest Ethiopia. (TIFF)

S4 Fig Picture of snails collected for a study of intestinal helminthiasis survey with emphasis on schistosomiasis in Koga irrigation scheme environs, northwest Ethiopia. (TIFF)

S5 Fig Picture of snails collected for a study of intestinal helminthiasis survey with emphasis on schistosomiasis in Koga irrigation scheme environs, northwest Ethiopia. (TIFF)
