# Supplementary material for: Intestinal helminthiasis survey with emphasis on schistosomiasis in Koga irrigation scheme environs, northwest Ethiopia
Source: PLoS One. 2022 Aug 8;17(8):e0272560. doi: 10.1371/journal.pone.0272560 (PMC9359581; doi:10.1371/journal.pone.0272560)
Supplement: S1 File — (PDF) [file pone.0272560.s001.pdf]

Questionnaire for a study on intestinal helminthiasis survey with emphasis on schistosomiasis in Koga irrigation scheme environs, Northwest Ethiopia.

Identification No.________________

Direction: Check the appropriate option or enter numbers or write the appropriate phase as required

001. Attending school name
  1. Mengesha Jemberie Primary School
  2. Merawi Primary School
  3. Wotet Abay Primary School

002. Sex of the student
  1. Male
  2. Female

003. Age of the student (years, months):_________________

004. Religion of the student
  1. Orthodox
  2. Muslim
  3. Protestant
  4. Others(specify)_______

005. Mother's education:
  1. Illiterate
  2. Read and rite
  3. Primary education
  4. Secondary and above

006. Father's education
  1. Illiterate
  2. Read and rite
  3. Primary education
  4. Secondary and above

007. Mother's occupation
  1. House wife
  2. Merchant
  3. Government employee
  4. Private employee

008. Father's occupation
  1. House wife
  2. Merchant
  3. Government employee
  4. Private employee

009. Residence of the student (specify kebele name)
  1. Urban: Kebele name _______
  2. Rural: Kebele name ________

010. Grade level of the student: _________________

011. Ethnicity of the student

1. Amhara
2. Agew
3. Others(specify): ___________________

012. Do you wear shoes?

1. Never
2. Sometimes
3. Always

013. If the response to question#107 is 2 or 3, what type of shoes do you wear?

1. Open shoes
2. Closed shoes

014. Water source for student's household

1. Tap water
2. Protected dug well
3. Protected spring
4. Unprotected spring

015. Adequacy of school latrine as per the standard:

1. Not adequate
2. Adequate

016. Do you use the school latrines?

1. Never
2. Sometimes
3. Always

017. For those replied '1' or '2' for Q #016; why don't you use it always?

1. Not clean
2. Not enough
3. Feeling uncomfortable

018. For those replied '1' or '2' for Q #017; where do you defecate instead?

1. Latrine outside the school compound
2. Openly in the bush around the school

019. Water source for school

1. Tap water
2. Protected dug well

020. Frequency of water supply interruption in school

1. Never
2. Rarely
3. about 1-2 days/week
4. three and above days/week

021. School had handwashing facility near toilets

1. No
2. Yes

022. Handwashing after meal

1. Never
2. Sometimes
3. Always

023. Handwashing after defecation

1. Never
2. Sometimes
3. Always

024. Do you go to the nearby school stream or river?

1. Never 2. Sometimes 3. Always

025. For those who replied ‘2 or 3’, do you have contact to river/stream water body?

1. Never 2. Sometimes 3. Always

026. What type of water contact do you have? (You can select more than one response)

1. Cross waterbody barefooted
3. Bathing
2. playing with water
4. Swimming

027. Have you ever taken medicine for intestinal parasites?

1. No 2. Yes

028. For those replied ‘Yes’, to Q#23:

1. from nearby health facility 2. school based deworming

029. For those replied ‘2’ to Q#024, when do you take the last school based deworming?

1. Within the last six months 2. Before a year
