# Supplementary material for: Intestinal helminthiasis survey with emphasis on schistosomiasis in Koga irrigation scheme environs, northwest Ethiopia
Source: PLoS One. 2022 Aug 8;17(8):e0272560. doi: 10.1371/journal.pone.0272560 (PMC9359581; doi:10.1371/journal.pone.0272560)
Supplement: S2 File — (PDF) [file pone.0272560.s002.pdf]

የአንጀት ጥገኛ ትላትሎች በተለይም የቢልሃርዚያን ስርጭት በሰሜን ምዕራብ ኢትዮጵያ ቆጋ የመስኖ ልማት አካባቢ ለማጥናት የተዘጋጀ መጠይቅ

መለያ ቁጥር.________________

መመሪያ: ትክክለኛዉን ምርጫ ምልክት ያድርጉ፤ ቁጥሩን ያስገቡ ወይንም ትክክለኛዉን ሃረግ ይፃፉ

001. የሚማሩበት ትምህርት ቤት ስም

1. መንገሻ ጀምበሬ የመጀመሪያ ደረጃ ት/ቤት
2. መራዊ የመጀመሪያ ደረጃ ት/ቤት
3. ወተት ዓባይ የመጀመሪያ ደረጃ ት/ቤት

002. ፆታ

1. ወንድ
2. ሴት

003. እድሜ(ኣመት, ወር):__________________

004. ሃይማኖት

1. ኦርቶዶክስ
2. እስልምና
3. ፕሮቴስታንት
4. ሌላ(ይጥቀሱ)________

005. የእናት የትምህርት ደረጃ:

1. ምንም ያልተማሩ
2. ማንበብና መፃፍ
3. የመጀመሪያ ደረጃ
4. ሁለተኛ ደረጃና በላይ

006. የአባት የትምህርት ደረጃ:

1. ምንም ያልተማሩ
2. ማንበብና መፃፍ
3. የመጀመሪያ ደረጃ
4. ሁለተኛ ደረጃና በላይ

007. የእናት ስራ

1. የቤት እመቤት
2. ነጋዴ
3. የመንግስት ሰራተኛ
4. የግል ስራ

008. የአባት ስራ

1. ገበሬ
2. ነጋዴ
3. የመንግስት ስራ
4. የግል ስራ

009. አድራሻ

1. ከተማ________
2. ገጠር

010. የክፍል ደረጃ: __________________

011. ብሄር

1. አማራ
2. አገዉ

3. ሌላ(ይጠቀስ): ____________________

012. ጫማ የመልበስ ልምድ አለህ/ሽ?

1. የለም   2. አልፎ አልፎ   3. ሁልጊዜ

013. ለጥያቄ#012፤ 2 ወይም 3 ለመለሱ, ምን አይነት ጫማ ነዉ የሚጫሙት?

1. ክፍት   2. ሽፍን

014. በቤት ዉስጥ የሚጠቀሙት ዉሃ

1. የቧንቧ ዉሃ
2. ያልተጠበቀ የጉድጓድ ዉሃ
3. የተጠበቀ የጉድጓድ ዉሃ
4. ያልተጠበቀ የምንጭ ዉሃ

015. የትምህርት ቤቱ መፀዳጃ ለተማሪዎቹ ቁጥር በቂ ነዉ? (በስታንዳርዱ መሰረት):

1. በቂ አየደለም   2. በቂ ነዉ

016. የትምህርት ቤታችሁን መፀዳጃ ቤት ይጠቀማሉ?

1. አልጠቀምም   2. አልፎ አልፎ   3. ሁልጊዜ

017. ለጥያቄ #016 መልሳቸዉ 1 ወይም 2 ለመለሱ'፤ ለምንድን ነዉ የማይጠቀሙት?

1. ንፁህ ስላልሆነ
2. በቂ ስላልሆነ
3. ነፃነት ስለማይሰማኝ

018. ለጥያቄ #016 መልሳቸዉ 1 ወይም 2 ለመለሱ; የት ነዉ የሚፀዳዱት?

1. ከትምህርት ቤት ዉጭ ካለ መፀዳጃ ቤት
2. ዉጭ ላይ፤ ጫካ ዉስጥ

019. የትምህርት ቤት ዉሃ አቅርቦት

1. የቧንቧ ዉሃ   2. የተጠበቀ የጉድጓድ ዉሃ

020. በትምህርት ቤት ዉሃ የመቆራረጥ ሁኔታ

1. የለም
2. አንዳንዴ
3. 1-2 ቀን በሳምንት
4. ከ 3 ቀን በላይ በሳምንት

021. ሽንት ቤት አካባቢ የእጅ መታጠቢያ አለ

1. የለም   2. አለ

022. ከምግብ በሁዋላ እጅ የመታጠብ ልምድ

1. የለም   2. አልፎ አልፎ   3. ሁልጊዜ

023. ከተፀዳዱ በሁዋላ እጅ የመታጠብ ልምድ

1. የለም   2. አልፎ አልፎ   3. ሁልጊዜ

024. ከትምህርት ቤታችሁ አቅራቢያ ወዳለዉ ወንዝ/ምንጭ የመሄድ ልምድ አለህ/ሽ?

1. የለም   2. አልፎ አልፎ   3. ሁልጊዜ

025. ለጥያቄ #024 መልስዎ ‘2 ወይም 3’ ለመለሱት፤ ከወንዙ/ምንጩ ወሃ ንክኪ ታደርጋለህ/ሽ?

1. የለም
2. አልፎ አልፎ
3. ሁልጊዜ

026. ወንዙ/ምንጩ ዉሃ ያለህ/ሽ ንክኪ ምን አይነት ነዉ? (ከአንድ በላይ መምረጥ ይቻላል)

1. በባዶ እግሬ ወንዙን አቋርጣለሁ
2. የወንዙ ዉሃ ላይ እጫወታለሁ
3. እታጠባለሁ
4. እዋኛለሁ

027. ለአንጀት ጥገኛ ትላትል መድሃኒት ወስደህ/ሽ ታዉቃለህ/ሽ?

1. የለም
2. አዎ

028. ለጥያቄ #027 መልስዎ ‘አዎ’ ከሆነ: ከየት ነበር የወሰዱት?

1. ከህክምና ቦታዎች
2. ትምህርት ቤት ከተሰጠን መድሃኒት

029. ለጥያቄ #028 መልስዎ ‘2’ ከሆነ, ለመጨረሻ ጊዜ ከትምህርት ቤት መድሃኒት የወሰዱት መቼ ነበር?

1. በዚህ 6ወር ዉስጥ
2. ከአንድ ዓመት በፊት
