# Supplementary material for: Intestinal helminthiasis survey with emphasis on schistosomiasis in Koga irrigation scheme environs, northwest Ethiopia
Source: PLoS One. 2022 Aug 8;17(8):e0272560. doi: 10.1371/journal.pone.0272560 (PMC9359581; doi:10.1371/journal.pone.0272560)
Supplement: S3 File — (PDF) [file pone.0272560.s003.pdf]

አዲስ አበባ ዩኒቨርሲቲ
አክሊሉ ለማ ፓቶባዮሎጂ መካነ ጥናት
አዲስ አበባ ፡ ኢትዮጵያ
✉ 1176
Fax: 251-11-2755296

ADDIS ABABA UNIVERSITY
Aklilu Lemma Institute of Pathobiology (ALIPB)
Addis Ababa, ETHIOPIA
☎ 251-11-276-30-91/213-57-25
e-mail: aklilu.lemma@aau.edu.et

Aklilu Lemma Institute of Pathobiology Institutional Review Board

Ethical Clearance Certificate

Ref. No.: ALIPB IRB/53/2013/21
Date: April 20, 2021

Title of the Project: 'Studies on *Plasmodium falciparum* and *Schistosoma mansoni* co-infection in communities along Koga water dam, northwestern Ethiopia: epidemiologic and immunologic aspects'

PI: Zemenu Tamir

Recommendation of the ALIPB Institutional Review Board

Dear Zemenu,

The ALIPB IRB has reviewed your above mentioned research proposal and noted its scientific merit. The IRB would like to remind the student to submit progress reports of the work every 6 months and the final report upon completion of the study. Furthermore, the student is expected to notify the ALIPB/IRB ahead of time any amendments or modifications in the protocol or premature suspension or termination of the study.

STATUS: APPROVED

Needs NRERC clearance: **Yes: ___ No: X**

IRB Chairperson: **Tilahun Teklehaymanot, Prof.**
Signature: ______ 20/04/2021

IRB Secretary: **Lemu Golassa, PhD**
Signature: ______ 20 04 2021

Approval

Name: **Dr. Mengistu Legesse, Director**
Signature: ______
Date: 20/04/2021

Cc// IRB office
