# Supplementary material for: Intestinal helminthiasis survey with emphasis on schistosomiasis in Koga irrigation scheme environs, northwest Ethiopia
Source: PLoS One. 2022 Aug 8;17(8):e0272560. doi: 10.1371/journal.pone.0272560 (PMC9359581; doi:10.1371/journal.pone.0272560)
Supplement: S1 Table — (PDF) [file pone.0272560.s005.pdf]

S1 Table. Association of sex and age with water contact characteristics of school aged children among study participants in Northwest Ethiopia, N=421.

		**Water contact**		**P-value**
Variable	Category	Yes, n(%)	No, n(%)	
Sex	Female	112(60.2)	74(39.8)	0.005
	Male	173(73.6)	62(26.4)	
Age in years	5-10	118(59.3)	81(40.7)	0.001
	11-15	167(75.2)	55(24.8)	
