# Supplementary figures and images for: Intestinal helminthiasis survey with emphasis on schistosomiasis in Koga irrigation scheme environs, northwest Ethiopia
Source: PLoS One. 2022 Aug 8;17(8):e0272560. doi: 10.1371/journal.pone.0272560 (PMC9359581; doi:10.1371/journal.pone.0272560)

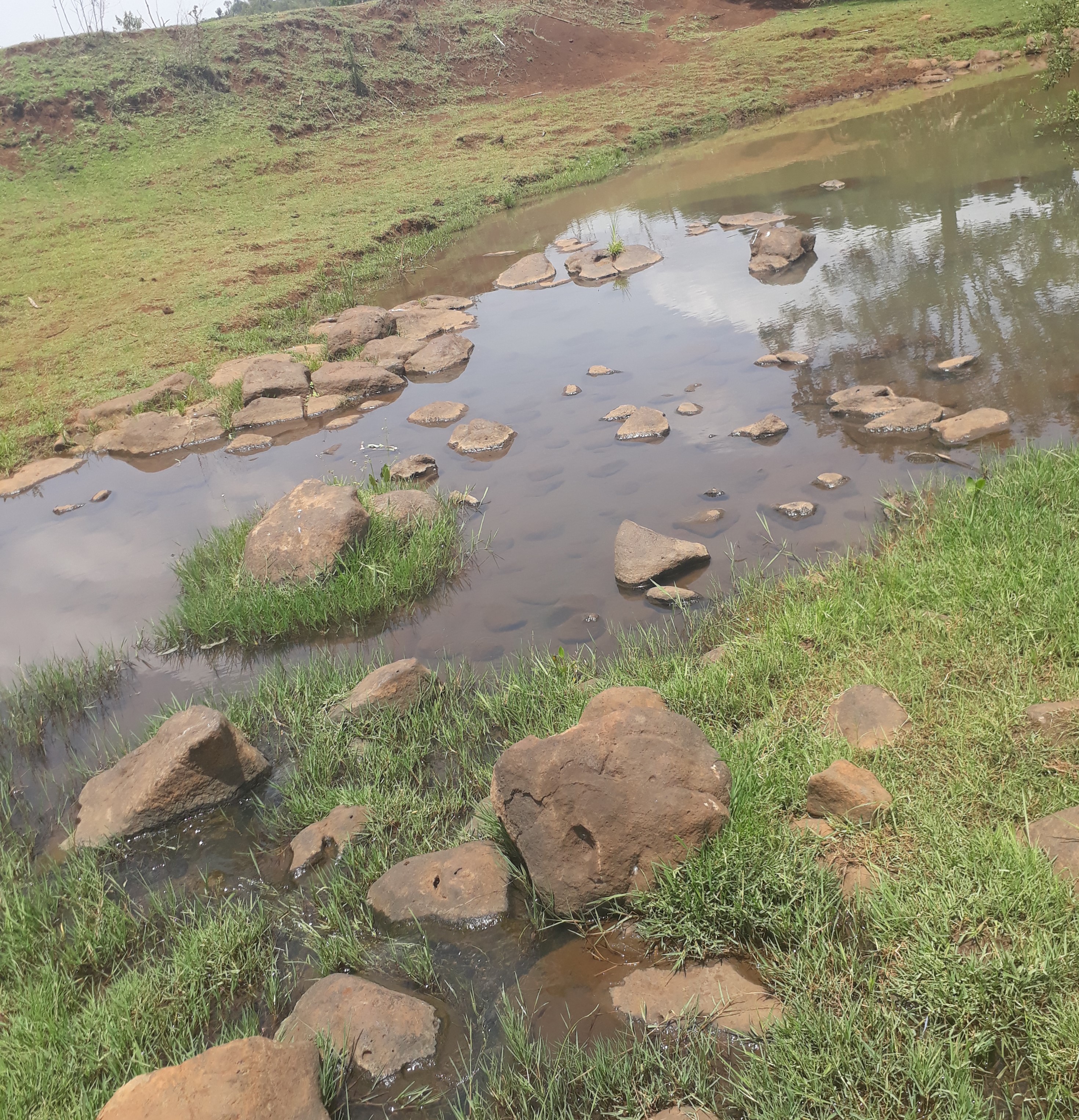

Supplement: S1 Fig — (TIFF) [file pone.0272560.s006.tiff]

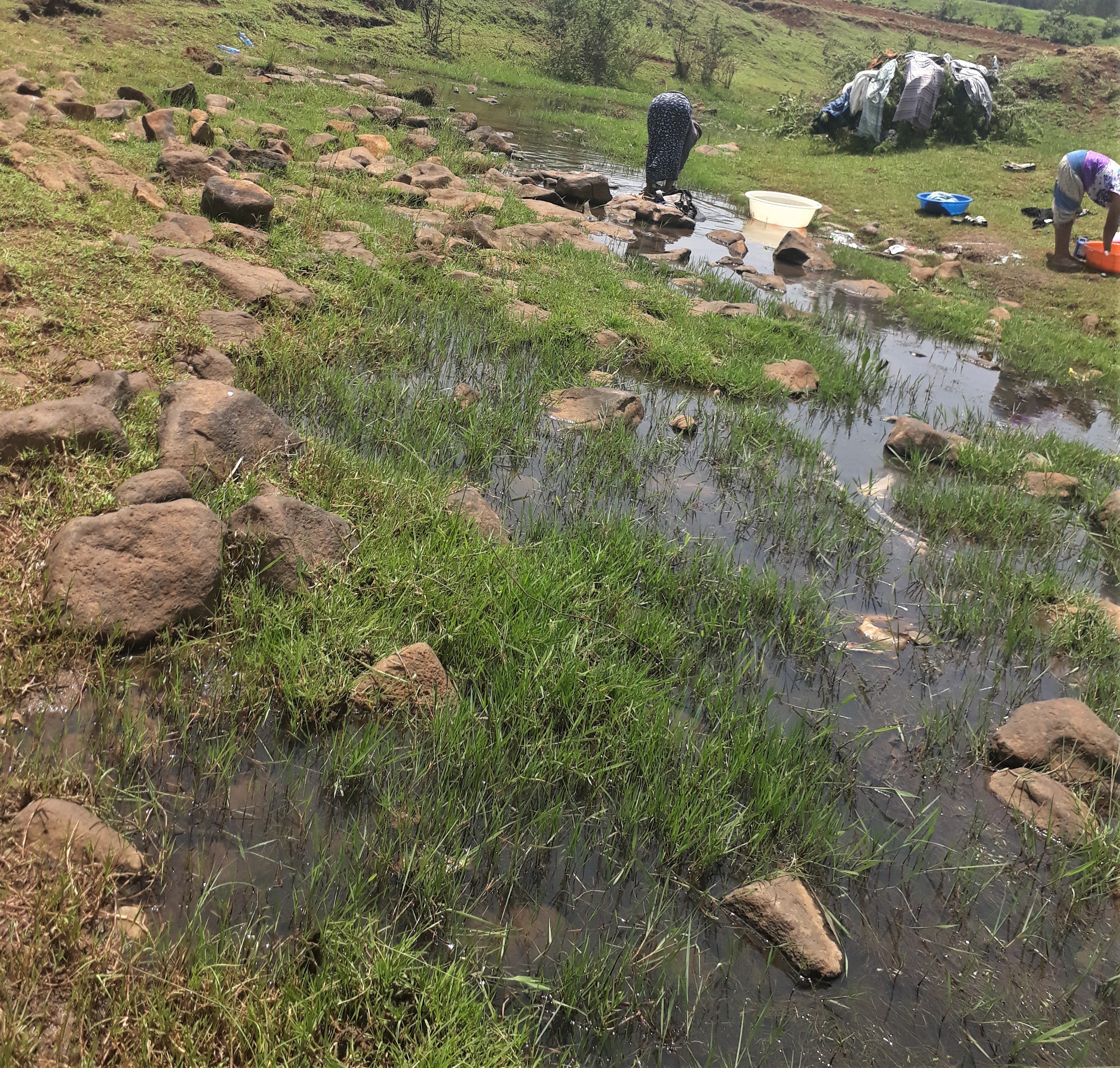

Supplement: S2 Fig — (TIFF) [file pone.0272560.s007.tiff]

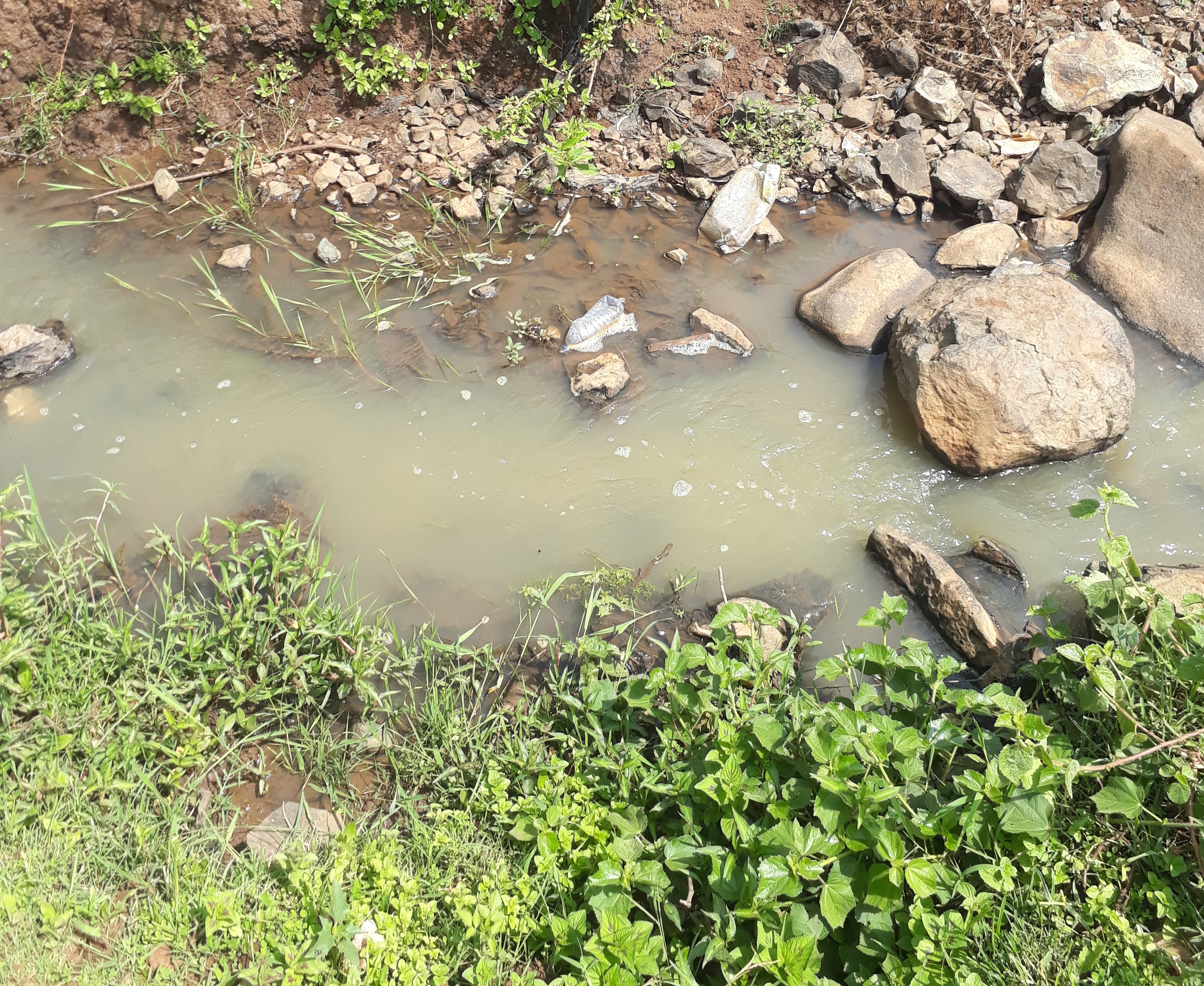

Supplement: S3 Fig — (TIFF) [file pone.0272560.s008.tiff]

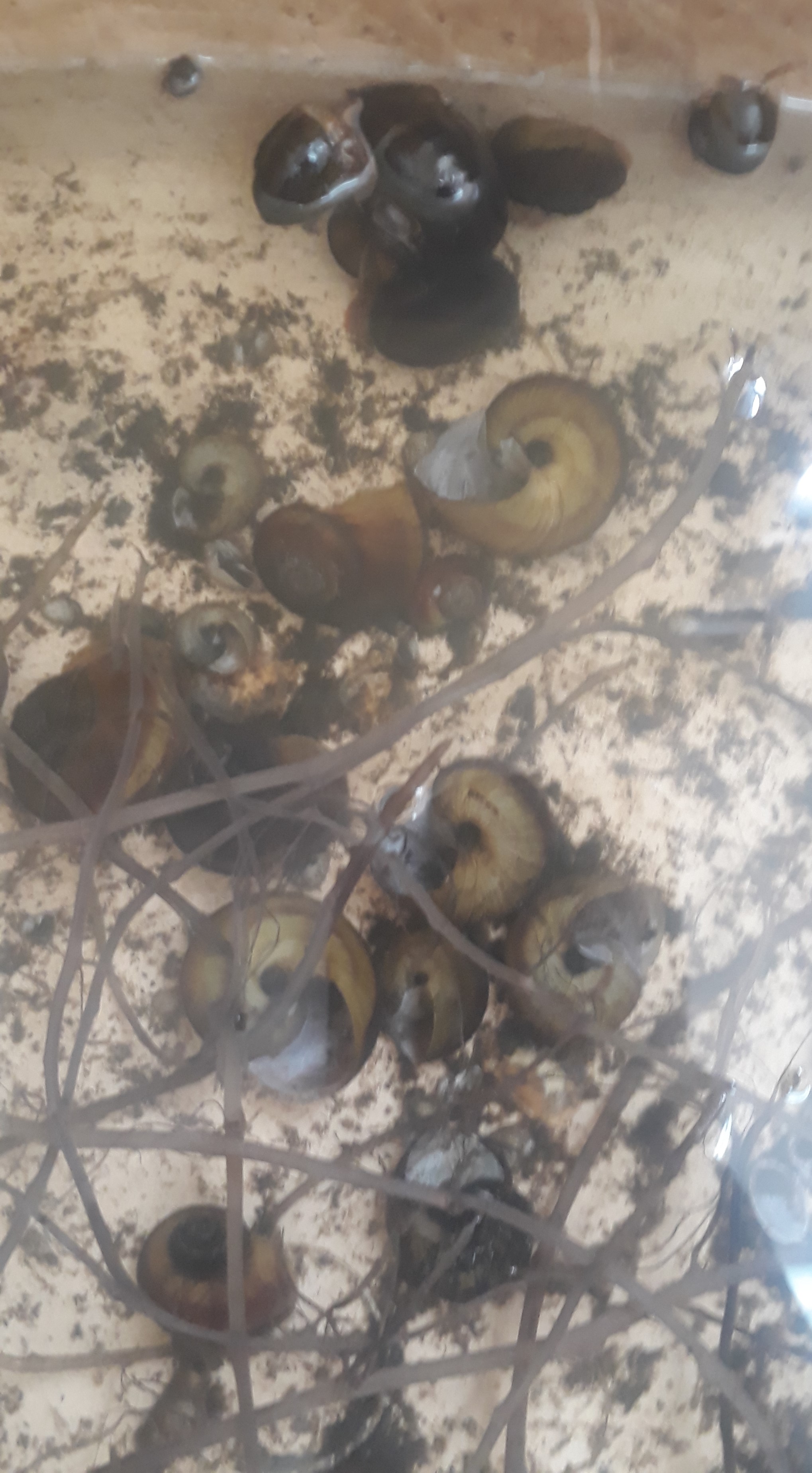

Supplement: S4 Fig — (TIFF) [file pone.0272560.s009.tiff]

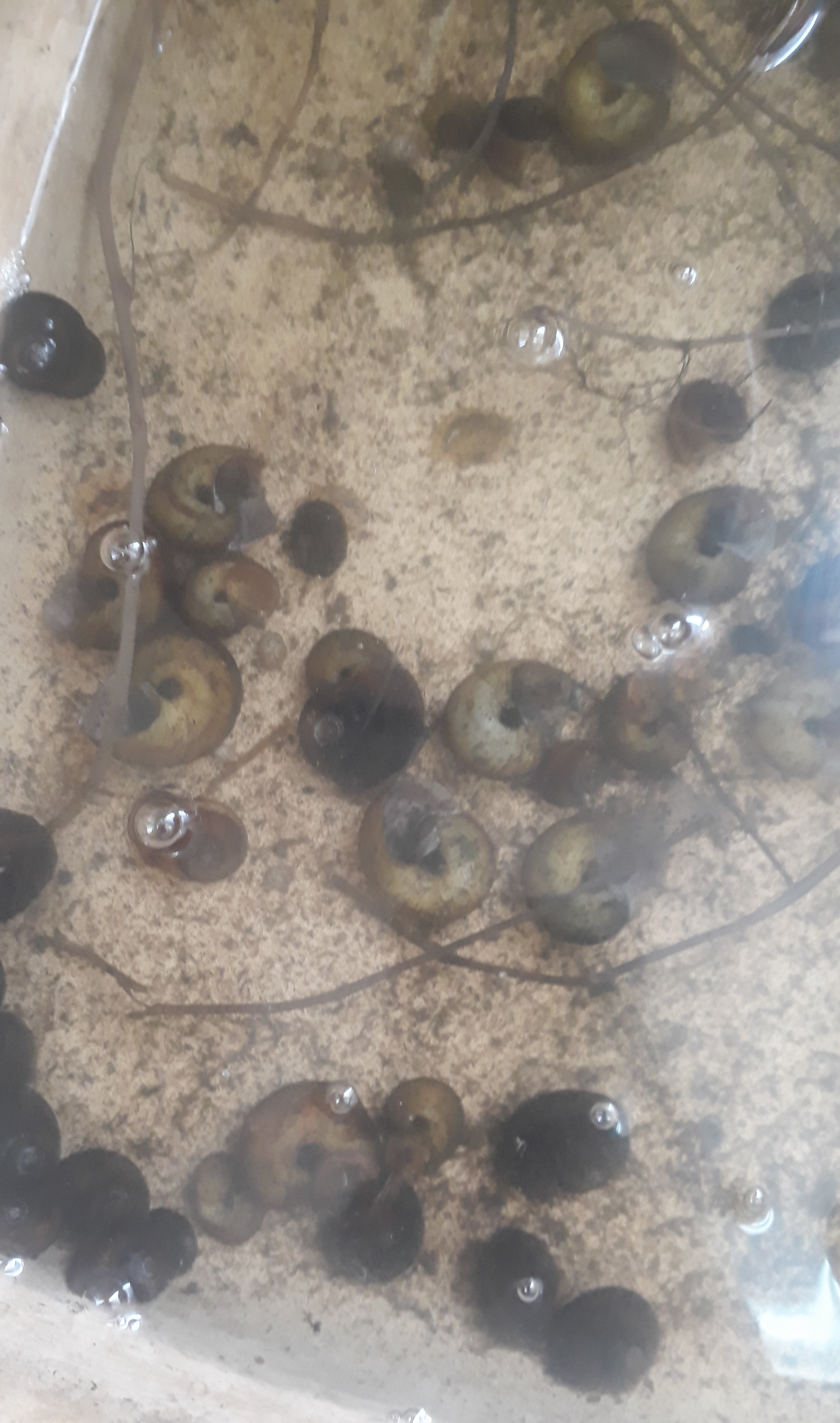

Supplement: S5 Fig — (TIFF) [file pone.0272560.s010.tiff]
